# The incidence of induced abortion in Kinshasa, Democratic Republic of Congo, 2016

**DOI:** 10.1371/journal.pone.0184389

**Published:** 2017-10-02

**Authors:** Sophia Chae, Patrick K. Kayembe, Jesse Philbin, Crispin Mabika, Akinrinola Bankole

**Affiliations:** 1 Guttmacher Institute, New York, New York, United States of America; 2 Department of Epidemiology & Biostatistics, University of Kinshasa School of Public Health, Kinshasa, Democratic Republic of Congo; 3 Department of Population Sciences and Development, University of Kinshasa, Kinshasa, Democratic Republic of Congo; National Academy of Medical Sciences, NEPAL

## Abstract

**Background:**

In the Democratic Republic of Congo, the penal code prohibits the provision of abortion. In practice, however, it is widely accepted that the procedure can be performed to save the life of a pregnant woman. Although abortion is highly restricted, anecdotal evidence indicates that women often resort to clandestine abortions, many of which are unsafe. However, to date, there are no official statistics or reliable data to support this assertion.

**Objectives:**

Our study provides the first estimates of the incidence of abortion and unintended pregnancy in Kinshasa.

**Methods:**

We applied the Abortion Incidence Complications Method (AICM) to estimate the incidence of abortion and unintended pregnancy. We used data from a Health Facilities Survey and a Prospective Morbidity Survey to determine the annual number of women treated for abortion complications at health facilities. We also employed data from a Health Professionals Survey to calculate a multiplier representing the number of abortions for every induced abortion complication treated in a health facility.

**Results:**

In 2016, an estimated 37,865 women obtained treatment for induced abortion complications in health facilities in Kinshasa. For every woman treated in a facility, almost four times as many abortions occurred. In total, an estimated 146,713 abortions were performed, yielding an abortion rate of 56 per 1,000 women aged 15–49. Furthermore, more than 343,000 unintended pregnancies occurred, resulting in an unintended pregnancy rate of 147 per 1,000 women aged 15–49.

**Conclusions:**

Increasing contraceptive uptake can reduce the number of women who experience unintended pregnancies, and as a consequence, result in fewer women obtaining unsafe abortions, suffering abortion complications, and dying needlessly from unsafe abortion. Increasing access to safe abortion and improving post-abortion care are other measures that can be implemented to reduce unsafe abortion and/or its negative consequences, including maternal mortality.

## Introduction

The Democratic Republic of Congo (DRC) is slowly emerging from decades of armed conflict that has stunted national development and produced disastrous consequences for the health of its citizenry. The country is beginning to rebuild its infrastructure, including its health system, and effort is being made to improve sexual and reproductive health indicators [1–3]. Until recently, very little attention or resources were devoted to providing access to modern contraceptives, and this effort is yet to translate into substantial improvement in service utilization. For instance, use of modern contraceptives remains very low in the country (8% among married women) [4]. Even in Kinshasa, the capital of the DRC and the second most populous city in Sub-Saharan Africa [5], modern contraceptive prevalence is low (23% among married women) [4]. Yet, 73% of married women do not want a child soon or at all [6]. On average, women desire less than four children—wanted fertility rate (WFR) is 3.6 [4]. On the other hand, at the low level of contraceptive use, Kinshasa women have only slightly more than four children on average—total fertility rate (TFR) is 4.2 [4]. This strongly suggests that many women are regulating their fertility through recourse to abortion, many of which are likely unsafe given the restrictive legal and social context.

Surveys conducted in the 1990s and early 2000s provide evidence that women in Kinshasa frequently resorted to abortion in the past: at least 15% of ever-pregnant women admitted to having had at least one abortion in their lifetime [7, 8]. Several studies also suggest that adolescent girls are particularly affected [9, 10], which is not surprising given that the median age at first sex is 17 years, the median age at first marriage is 19 years, and 58% of unmarried sexually active women do not use modern contraception [6]. Additional evidence points to women resolving unwanted pregnancies through abortion, particularly using unsafe methods. Unsafe abortion, one of the leading causes of maternal mortality, contributes up to 10% of maternal deaths in Sub-Saharan Africa [11]. Indeed, it is likely that fatal abortion complications contribute substantially to the DRC’s high maternal mortality ratio, which is one of the highest in the world. In 2015, an estimated 693 maternal deaths occurred for every 100,000 live births. To put this into perspective, the DRC’s estimated ratio is 21% higher than the average estimate for Sub-Saharan Africa (546 per 100,000 live births), and is higher than the ratio for most of its neighbors, with the exception of Burundi, Central African Republic, and South Sudan [12].

The DRC’s penal code prohibits the provision of abortion altogether [13, 14]. In practice, however, it is widely accepted that this procedure can be performed to save the life of a pregnant woman [13, 15–17]. Still, it is still very difficult to obtain a legal abortion, even for a woman in need of a life-saving procedure. Thus, women who desire to terminate their pregnancy, have little or no choice but to seek illegal and often unsafe abortions. Given that most abortions occur clandestinely, its prevalence is difficult to measure. In this paper, we present estimates of the incidence of abortion in Kinshasa in 2016, using the Abortion Incidence Complications Method (AICM). We also present the incidence of unintended pregnancy and the proportion of unintended pregnancies that end in abortion. The AICM has been used across multiple developing countries where abortion is highly restricted and/or where official statistics are not compiled [18–20].

## Data and methods

Our study draws on multiple data sources to estimate measures of abortion incidence and unintended pregnancy. The authors conducted three surveys to collect the primary data used in this study: Health Facilities Survey (HFS), Health Professionals Survey (HPS), and Prospective Morbidity Survey (PMS). The HFS and PMS provide information on the annual number of women treated for abortion complications (i.e. received postabortion care (PAC)) at health facilities across Kinshasa while the HPS generates information that is used to calculate a multiplier representing the number of women who have abortions for every woman who has an induced abortion and receives facility-based treatment. To derive our estimates of induced abortions and unintended pregnancies, we also draw on other data sources, including official government statistics and the 2013–2014 Democratic Republic of the Congo Demographic and Health Survey (DRC DHS), for Kinshasa-specific population and poverty estimates, age-specific fertility rates, and the proportion of women who deliver in health facilities [21, 22]. We obtained approval to carry out this study from the institutional review boards (IRBs) of the Guttmacher Institute and the University of Kinshasa, School of Public Health.

### Health Facilities Survey

The study team compiled a list of 2,713 health facilities in Kinshasa whose level of equipment and staffing make them likely to provide PAC. Although the Ministry of Health possesses an official list of registered health facilities, many facilities in Kinshasa are not registered with the government. Thus, the study team combined the Ministry of Health’s official list with two listings conducted for other research projects that members of the study team have been involved in. After combining the three lists, we removed all duplicate facilities that appeared on the combined list. The compiled list included information on level of facility and ownership type (public, private, and non-government organization (NGO)). In the public sector, four levels of facilities are capable of treating abortion complications: university hospital, provincial hospital, general reference hospital/reference health centers, and health centers. In the private/NGO sector, two levels of facilities exist: hospitals and health centers. We removed all specialist facilities that would not be expected to provide PAC such as pediatric, dental, and eye clinics.

To obtain a representative sample of facilities for Kinshasa, we used stratified random sampling to select 423 facilities. We first split the facilities by their designated levels (i.e. different types of hospital and health center) and then stratified by ownership type (public, private/NGO). We collapsed private and NGO facilities into the same category due to difficulties in distinguishing between the two types. Since we expected hospitals to provide a large proportion of postabortion care and they are generally smaller in number, we included 100% of hospitals in our sample. Given that health centers typically do less PAC and they are usually large in number, we normally select a small fraction of them for inclusion in the sample, the larger the total number the smaller the proportion. We initially planned to include 10% of health centers, regardless of ownership type, in our sample, but due to the low number of public health centers, we opted to oversample facilities in this facility type. Thus, we randomly selected 19% of the public health centers and 10% of the private and NGO health centers.

In each of the facilities in our sample, a senior staff member who would be knowledgeable about the facility’s PAC services was interviewed after obtaining his/her consent to participate in the survey. In larger facilities, such as hospitals, the chief of the obstetrics and gynecology department or an obstetrician-gynecologist was usually selected. In smaller facilities, such as health centers, this person was typically the director or another provider (e.g. nurse or midwife). Each consenting staff member completed a face-to-face interview using a structured questionnaire. The respondent was asked if the health facility treated complications from abortion, either spontaneous or induced, that were serious enough to require treatment in a health facility. If the respondent reported that the facility provided this service, the interviewer asked for the number of women treated for abortion complications as outpatients and as inpatients in an average month and in the past month. If the respondent was unable to provide these numbers, he or she was asked to provide the number of women treated in the average year and past year. We requested estimates for two time frames to capture variability that might exist in monthly or yearly caseloads, as abortion, especially induced abortion may be seasonal in some contexts [23, 24]. For facilities that provided PAC estimates for the average month or past month, we multiplied these estimates by 12 to generate annual estimates.

Fieldwork for the HFS was conducted in April and May 2016. The survey team successfully conducted interviews at 361 out of 423 facilities in our sample, resulting in an overall response rate of 85% (Table 1). Response rates varied by facility type, and ranged from 83% of private/NGO health centers to 100% among the university hospital, provincial hospital, and public health centers. Non-response was primarily due to interviewers finding facilities no longer in operation or impossible to locate. During the interviews, the survey team learned that several facilities in our sample were misclassified, either by level of facility (hospital or health center), ownership (public or private/NGO), or both. Because the PAC caseloads of these misclassified facilities were similar to those of facilities using the pre-fieldwork classification of facility type, we did not reclassify them for the purposes of constructing sampling weights. We did, however, use the post-fieldwork classification of health facilities when estimating PAC caseloads by facility level and/or ownership. We constructed sampling weights using information on the proportion of facilities selected into the sample and the response rates by level of facilities and ownership. We assumed that the average PAC caseload in a specific facility type did not differ between sampled and non-sampled facilities. By applying the weights to the data, we generated estimates of PAC caseloads for all facilities in Kinshasa.

**Table 1:** Survey response and PAC provision by facility type, Health Facilities Survey and Prospective Morbidity Survey, Kinshasa 2016.

	Health Facilities Survey			Prospective Morbidity Survey		
Facility type	Number of sampled facilities	Number of sampled facilities completing interview (%)	Number of interviewed facilities providing PAC (%)	Number of eligible facilities participating in PMS (%)^a^	Median annual PAC caseload per facility	Interquartile range of annual PAC caseload per facility
University hospital (public)	1	1 (100)	1 (100)	1 (100)	60	0
Provincial hospital (public)	1	1 (100)	1 (100)	1 (100)	252	0
Public other hospitals	37	36 (97)	31 (86)	31 (100)	72	48
Private/NGO other hospitals	99	84 (85)	79 (94)	67 (85)	36	48
Public health centers	19	19 (100)	11 (58)	9 (82)	36	18
Private/NGO health centers	266	220 (83)	139 (63)	114 (82)	30	30
**Total**	**423**	**361 (85)**	**262 (73)**	**223 (85)**	**36**	**42**

Note: Facility type refers to the category identified post-fieldwork.

^a^ All facilities that reported providing PAC in the Health Facilities Survey were eligible to participate in the Prospective Morbidity Survey.

### Prospective Morbidity Survey

The study team conducted the Prospective Morbidity Survey (PMS) after HFS data collection was completed. All facilities that reported providing PAC in the HFS were eligible to participate in the PMS. The PMS consisted of two parts, patient survey and provider survey. The type of information collected differed by type of survey. While the patient survey collected information on the characteristics of women obtaining PAC and the conditions under which abortions take place, the provider survey focused on the severity and management of abortion complications. The study team invited each of the eligible health centers to send one staff member and each of the hospitals two staff members to participate in a three-day interviewer training. Clinic staff were recruited to serve as interviewers in their respective facilities because they were in a position to know when PAC patients were being treated and to be able to determine the appropriate time to conduct interviews with consenting respondents and their primary care providers.

Interviewers attempted to conduct interviews with all women treated for abortion complications, spontaneous or induced, over a 30-day period. All women, regardless of whether they were treated as inpatients or outpatients, were eligible to be included in the study. Interviewers approached patients once they were in stable condition and sought informed consent to conduct the interview. If an interviewer was the patient’s primary care provider, he or she was not permitted to interview the patient and had to ask another interviewer working in the facility (if there was one) or ask his or her supervisor to conduct the interview. The study team restricted interviewers from interviewing their patients to reduce any concern that patients might feel about their responses affecting their treatment. After the interview, the interviewer asked the respondent for her consent to interview the patient’s health provider. If the patient gave her consent, then the interviewer approached the patient’s provider to obtain informed consent and carry out the interview.

For various reasons, not all eligible patients were interviewed in the PMS. Interviewers filled out a tracking sheet that kept track of missed cases and the primary reason they were missed. Reasons for missed cases included: refusal to participate; interviewer not present at the facility when the respondent was there; interviewer was the patient’s primary care provider and another interviewer or supervisor was unavailable; patient transferred to another facility for further treatment; too sick to participate; or died. By keeping track of missed cases, we determined the total number of women treated for abortion complications during the 30-day study period, regardless of whether they were interviewed. For each health facility, we determined the facility’s PAC caseload during a 30-day period by adding the number of women interviewed to the number of missed cases. However, not all women who obtain PAC in health facilities across Kinshasa necessarily need treatment, particularly those who induced their abortions using misoprostol. Though HFS respondents were instructed not to include abortion complications that would have been resolved on their own (without any care) in their counts of PAC patients, these instructions were not given in the PMS. Thus, the PMS possibly collected data from women whose abortion complications did not necessarily need treatment. To make the PMS caseload consistent with the caseload captured in the HFS, we subtracted women who likely did not need treatment. According to the data collected in the PMS, at least 5% of PAC patients had misoprostol-induced abortions, experienced no complications, and were discharged in good health in less than 24 hours. We assumed that these women were in the process of completing their abortions and would not have needed PAC. We subtracted these women from the total number of PAC cases treated in health facilities. Similar to estimates produced using the HFS, we multiplied the 30-day caseload by 12 to determine the annual caseload for each health facility.

Among the 262 facilities eligible to participate in the PMS, 223 participated, resulting in a response rate of 85%. Refusal to participate was the most common reason for non-response; during the period between the HFS and PMS, four facilities eligible for the PMS closed down. Fieldwork for the PMS took place in July and August 2016.

### Health Professionals Survey

The study team compiled a list of professionals who are knowledgeable about the conditions of abortion provision and post-abortion care in Kinshasa. This list, which was compiled in consultation with colleagues working in different domains of reproductive health, including research, policy, and community health programs, consisted of medical doctors, nurses, researchers, policymakers, advocates, social workers, NGO staff, and other individuals who would be well-informed about women’s behaviors and outcomes in seeking and obtaining abortion. Approximately two in five respondents were clinicians (doctors, nurses, midwives) and the rest were non-clinicians.

The primary purpose of the Health Professionals Survey was to collect information on: 1) distribution of abortions by method used (surgical, misoprostol, and others) 2) distribution of women having abortions by type of method used and by type of provider (doctors, nurses or midwives, traditional practitioners, pharmacists, self-induction, and other untrained persons) 3) proportion of women experiencing complications (defined as health problem resulting from an abortion and serious enough to require treatment in a health facility) by type of method used and by type of provider and 4) proportion of women experiencing complications who are likely to obtain care at a health facility by type of method used. Because women’s access to abortion, particularly provider types and methods of abortion, likely varies by women’s socioeconomic status, respondents were asked to provide responses separately for poor women and non-poor women. In total, interviews were conducted with 115 respondents. Data from two respondents were not analyzed because they did not provide responses to any of the key questions. Fieldwork for the HPS was conducted from May to August 2016.

### Analysis

Several steps were taken to estimate the incidence of abortion and unintended pregnancy in Kinshasa.

#### Calculating the PAC caseload in health facilities

We used data collected in the HFS and PMS to estimate the PAC (induced or spontaneous) caseload in health facilities across Kinshasa. Though the HFS collected data on PAC caseloads for the past month/year and average month/year, we chose not to use the past month/year estimate because it was considerably lower than both the average month/year estimate and the PAC caseload estimated from the PMS, which was based on prospective reporting over a period of one month. In the case of HFS estimates for the average month, respondents report retrospectively and provide an average monthly caseload based on their experience. The PMS estimate was slightly higher than the HFS average year estimate. One possible explanation is that PAC caseloads were underestimated in the HFS. HFS respondents were asked to rely on their memory when reporting PAC caseloads for the average month or average year. Consequently, recall bias may have affected estimates. The PMS, in contrast, does not suffer from the effects of recall bias because interviewers attempted to conduct interviews with all women treated for abortion complications prospectively as they were admitted for treatment, over a 30-day period. Even if all eligible women were not interviewed, interviewers kept track of the number of missed cases and reported them to the survey team at the end of the observation period. Alternatively, PAC caseloads could have been overestimated in the PMS. Health facility workers, who served as interviewers, were financially remunerated for each pair of patient and provider interviews conducted during the study period. It is, therefore, possible that some interviewers fabricated cases to receive the incentive. While this cannot be ruled out, we expect it to be minimal because supervisors were instructed to verify the existence of each case.

If a health facility participated in both the HFS and PMS, we averaged the two data points to obtain an average caseload for that facility. Otherwise, we used the caseload for the average year as the average caseload for that facility. In total, 35 health facilities did not participate in the PMS.

Some women who received PAC in a heath facility were referred to another facility, usually a higher-level facility, for additional care. As a result, these women would have sought treatment in two facilities, and would be double-counted in estimates of PAC provision. HFS respondents were asked to estimate the number of women who received PAC in their facility and then referred to another facility for additional treatment in the past month or year. If the number of referrals were given for the past month, we multiplied this number by 12 to produce an estimate for the past year. Across all facilities in Kinshasa, we calculated that 5,606 referrals were made in the past year. Dividing the number of referrals by the total number of post-abortion cases (32,590) treated in the past year, we determined that 17.2% of all PAC cases were referred to another facility. Not all women referred to another facility necessarily seek treatment, but because no data exists on the percentage of women referred for PAC who seek treatment, we assumed that the percentage seeking treatment is similar to the percentage of women in Kinshasa delivering in health facilities, which according to the most recent DRC DHS is 98% [4]. By multiplying the percentage of referral cases (17.2%) by the percentage of referrals assumed to have sought treatment (98%), we calculated that 16.9% of PAC cases needed to be subtracted from the total number of PAC cases treated in health facilities in Kinshasa.

The objective of this study is to estimate the incidence of abortion in Kinshasa. Given that Kinshasa has a high density of health facilities, women from nearby areas may come to Kinshasa for PAC. Not taking this into account would overestimate the incidence of abortion. According to data collected in the PMS, 3% of women receiving PAC in health facilities in Kinshasa live outside the city. Thus, we subtracted 3% of PAC cases from the total number of abortion complications treated in health facilities in Kinshasa.

#### Subtracting the number of spontaneous abortion complications

To determine the number of complications that are due to induced abortions, we subtracted the number of complications resulting from spontaneous abortions from the total number of PAC cases. Similar to previous studies applying the AICM [18, 19, 25], we assumed that only women who have late miscarriages (between 13 and 22 weeks’ gestation) seek treatment at health facilities. Prior studies have shown that late miscarriages make up 3.4% of all live births [26, 27]. We estimated the number of late miscarriages by applying this proportion to the number of live births in Kinshasa, which was calculated by applying age-specific fertility rates from the 2013–14 DRC DHS to population estimates of women of reproductive age in Kinshasa.

Not all women who experience a late miscarriage seek treatment at health facilities. Similar to previous studies where the AICM has been applied [18, 20, 28], we assumed that the proportion of women seeking PAC for late miscarriages is similar to the proportion of women giving birth in hospitals (98%).

#### Calculating the multiplier

We used data collected in the HPS to determine the proportion of women having induced abortions who receive PAC. The HPS provided estimates of: 1) the proportion of women who experience complications by type of abortion method and type of abortion provider and 2) the proportion of women who obtain PAC by type of abortion method. These estimates were calculated separately for poor and non-poor women because these subgroups of women may have different abortion-seeking and treatment behaviors. We used this information to calculate the proportion of poor and non-poor women seeking treatment for induced abortion complications. Next, we weighted these proportions by the distribution of women of reproductive age in Kinshasa by poor and non-poor status to come up with the proportion of all women who have abortions who will receive treatment in health facilities. The inverse of this proportion is our multiplier or adjustment factor. This multiplier represents the number of women who have abortions for every woman who has an abortion and obtains PAC in a facility. A mathematical expression of the inputs and steps for calculating the multiplier can be found in S1 File.

#### Estimating abortion measures

In our initial calculation of the number of abortion complications, spontaneous or induced, treated in facilities, we obtained 95% confidence intervals around the estimate. To estimate the total number of induced abortions that occur in Kinshasa in 2016, we applied the multiplier to the number of induced abortion complications treated in health facilities (after subtracting referrals treated, complications experienced by women who lived outside of Kinshasa, and cases of late term miscarriages presented in facility for care). This calculation was done for the point estimate of abortion complications, as well as its lower and upper bounds, since these were obtained from a random sample of health facilities. We applied this to the estimates of induced abortions to enable us to provide lower and upper estimates of the number of induced abortions in Kinshasa. We then calculated the abortion rate by dividing the number of induced abortions by the population of women of reproductive age (15–49 years), and the abortion ratio by dividing the number of induced abortions by the number of live births in Kinshasa. By dividing the lower and upper estimates of the number of induced abortions by the reproductive-age population, we also obtained lower and upper estimates for the induced abortion rate.

#### Estimating unintended pregnancy

We estimated the annual number of pregnancies in Kinshasa by summing the annual number of live births, induced abortions, and miscarriages. Prior studies have demonstrated that the number of miscarriages is equal to the sum of 20% of live births and 10% of induced abortions [26, 27]. Thus, we calculated the number of unintended pregnancies in the following manner: Unintended pregnancies equal induced abortions plus unplanned births plus miscarriages resulting from unintended pregnancies (calculated as the sum of 20% of unplanned births and 10% of induced abortions). Unplanned births are defined as births that were unintended at the time of conception. Although we assumed that all induced abortions were unintended pregnancies, we recognize that some (usually a small number) intended pregnancies may have ended in induced abortions. We calculated the number of unplanned births by multiplying the proportion of births in Kinshasa reported to be unintended in the past five years in the 2013–14 DHS in the DRC by the estimated number of live births in the city. Similarly, the number of intended pregnancies equals planned births plus miscarriages of intended pregnancies (i.e. 20% of planned births). Planned births are defined as births that were intended at the time of conception. We calculated the number of planned births by multiplying the proportion of births in Kinshasa reported to be intended in the past five years in the 2013–14 DHS in the DRC by the number of live births in the city. We calculated the unintended pregnancy rate by dividing the number of unintended pregnancies by the population of women of reproductive age (15–49 years).

## Results

### Postabortion care

Variation exists in the average annual PAC caseloads by facility type (Table 1). The PAC caseload is highest at the provincial hospital, which is a public facility, and one of the largest hospitals in Africa. In 2016, this facility treated 252 women for abortion complications. Surprisingly, with the exception of the provincial hospital, average PAC caseloads do not differ by level of facility, varying instead by ownership type. Though fewer public facilities exist in Kinshasa than private/NGO facilities, the former treat a substantially higher PAC caseload per facility than do those in the private/NGO sector. The median annual caseload in other public hospitals (not university or provincial hospitals) is 72 while it is 36 in private/NGO hospitals. A similar pattern is observed among health centers: The median annual number of PAC cases treated per public health center is 36 while it is 30 per private/NGO health center.

**Fig 1:**
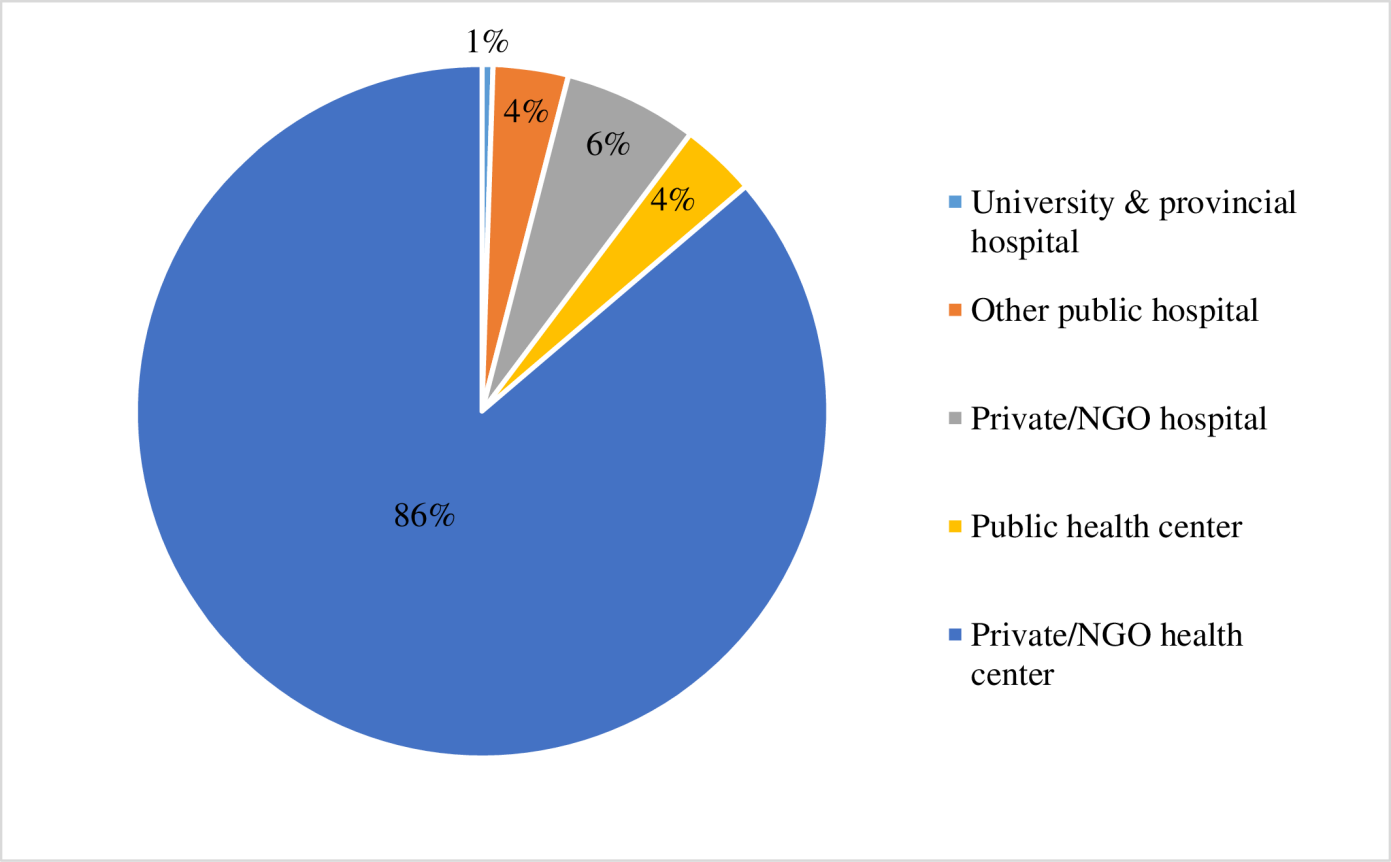
Percentage distribution of postabortion cases, by facility type, Kinshasa 2016.

Although median PAC caseloads are higher in public facilities, regardless of level of facility, the overwhelming majority of PAC, 90%, is provided by health centers in Kinshasa, and almost exclusively in the private/NGO sector (Fig 1). Hospitals, of which two-thirds are in the private/NGO sector, treated 10% of all PAC cases. Taken together, private/NGO hospitals and health centers treated 93% of all PAC cases; the remaining 7% were treated in public sector hospitals and health centers.

In 2016, an estimated 60,870 women received PAC in health facilities across Kinshasa (Table 2). From this total number of complications, we subtracted 23,004: 10,261 double-counted referral visits; 1,518 women from outside of Kinshasa; and 11,226 women treated for complications due to spontaneous abortions. The remaining 37,865 is the estimated number of women who received PAC for induced abortion complications, which translates to an induced abortion treatment rate of 14 per 1,000 women aged 15–49. According to these estimates, 77% of all PAC cases in Kinshasa were due to induced abortion.

**Table 2:** Number of women treated for abortion complications, Health Facilities Survey And Prospective Morbidity Survey, Kinshasa 2016.

Women treated for abortion complications (spontaneous or induced)	60,870
Double-counted referral cases	10,261
Women from outside of Kinshasa	1,518
Women treated for complications of spontaneous abortions	11,226
Women subtracted from total number of abortion complications	23,004
Women treated for induced abortion complications	37,865
Induced abortion treatment rate (per 1,000 women ages 15–49)	14
Percent of treated complications due to induced abortion	77

### Induced abortion and unintended pregnancy

Applying the multiplier (3.9) to the number of induced abortion complications treated, we estimated that 146,713 induced abortions took place in Kinshasa in 2016; our lower and upper estimates are 128,684 and 164,079 (Table 3). We calculated an induced abortion rate of 56 (lower: 49, upper: 62) per 1,000 women aged 15–49, and an abortion ratio of 44 per 100 live births.

**Table 3:** Estimates of induced abortion and unintended pregnancy in Kinshasa, 2016.

***Abortion measures***	** **
Number of women treated for induced abortion complications	37,865
Multiplier^a^	3.9
Number of induced abortions	146,713
Lower estimate	128,684
Upper estimate	164,079
Abortion rate (per 1,000 women ages 15–49)	56
Lower estimate	49
Upper estimate	62
Abortion ratio	44
***Unintended pregnancy measures***	** **
Number of pregnancies	563,064
Pregnancy rate (per 1,000 women ages 15–49)	241
Number of unintended pregnancies	343,085
Unintended pregnancy rate (per 1,000 women ages 15–49)	147
Percent of pregnancies that were unintended	61%
Percent of unintended pregnancies that ended in abortion	43%

^**a**^Multiplier is rounded to one decimal place.

In 2016, an estimated 563,064 pregnancies occurred in Kinshasa, resulting in a pregnancy rate of 241 per 1,000 women of reproductive age (Table 3). Unintended pregnancies were more common than intended pregnancies and often ended in abortion; 61% of all pregnancies were unintended and 43% of these pregnancies were aborted. We estimated an unintended pregnancy rate of 147 per 1,000 women aged 15–49.

**Fig 2:**
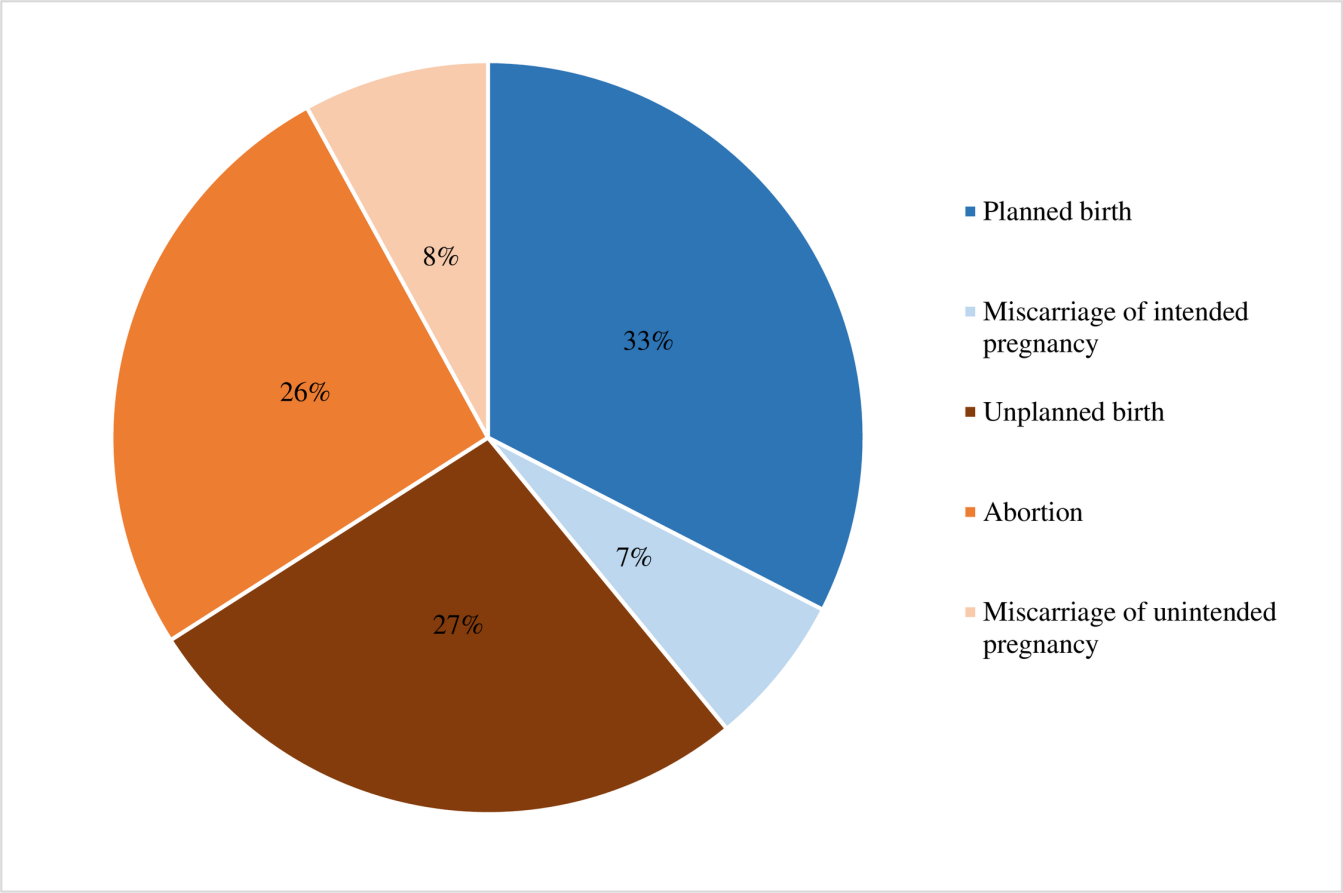
Percentage distribution of pregnancies by outcome and intention status, Kinshasa 2016.

Lastly, we calculated the percentage distribution of all pregnancies by intention status and outcome: 33% are planned births, 27% unplanned births, 26% induced abortions, 7% miscarriages from intended pregnancies, and 8% miscarriages from unintended pregnancies (Fig 2). Thus, the majority of pregnancies are unintended.

## Discussion

This article presents the first estimates of abortion incidence and unintended pregnancy in Kinshasa, DRC. We estimated that 146,713 induced abortions occur every year in Kinshasa, yielding an abortion rate of 56 per 1,000 women aged 15–49. We also calculated that more than 343,000 unintended pregnancies occur annually, resulting in an unintended pregnancy rate of 147 per 1,000 women aged 15–49. Taken together, these findings show that two in five unintended pregnancies end in abortion. Women in Kinshasa are clearly struggling to prevent unintended pregnancies, which often result in unwanted childbearing and abortion.

In Kinshasa, the overwhelming majority of abortion complications are treated in lower-level facilities, specifically private or NGO-affiliated health centers. This differs from the pattern that is typically observed in countries where the AICM has been applied: Most women obtain PAC at higher-level facilities such as hospitals [18, 29, 30]. There are several possible explanations for why PAC is mainly sought in health centers rather than hospitals in Kinshasa. A high concentration of doctors live and work in Kinshasa [31]. Though doctors may prefer to work in hospitals, there may not be enough hospitals to employ them. Rather than work in other parts of the country, doctors may choose to remain in Kinshasa and operate their own health centers. Additionally, there are many nurses trained in private training institutions that are unable to find employment and who also establish private health centers. As a result, many health centers across Kinshasa have the necessary staff to provide PAC. Another possible explanation is that abortion complications are not serious enough to warrant treatment in hospitals. As a result, women first seek treatment at health centers, and if complications are serious enough, are then referred to hospitals for treatment. Furthermore, although PAC is legal, the fact that abortion is forbidden outright in the DRC coupled with strong social stigma against abortion may cause women to prefer to seek care from private and NGO facilities, where confidentiality and humane treatment by providers are more likely guaranteed, than public facilities.

The abortion rate in Kinshasa (56 per 1,000 women aged 15–49) is higher than the 2010–2014 rate for Middle Africa (35 abortions per 1,000 women of reproductive-age) [32]. Given that Kinshasa is the capital city, it is probable that Kinshasa’s abortion rate is higher than the country’s rate. In other African countries where AICM studies have been conducted and abortion rates were calculated for the capital city, abortion rates were higher in the capital compared to the entire country [25, 33, 34]. For example, Rwanda’s abortion rate was 25 while Kigali’s rate was 87; Uganda’s abortion rate was 39 while Kampala’s rate was 77; and Senegal’s rate was 17 while Dakar’s rate was 21. Abortion rates are likely higher in capital cities for a number of reasons, including better access to abortion services, greater desire to have fewer children and relatively more liberal views about social issues in capital cities than in other parts of the country.

Emerging evidence from many parts of sub-Saharan Africa indicate that growing numbers of women are using misoprostol to induce abortions [35–38]. Findings from the PMS substantiated that this is also true in Kinshasa. When administered properly, misoprostol can be safely used to terminate a pregnancy [39–41]. Though it’s normal to experience bleeding after taking misoprostol [42], some women taking this medication without medical supervision may be concerned about the amount of bleeding they experience and seek treatment at health facilities [43]. Evidence also suggests that in some countries, untrained sellers of this drug do not give women adequate guidelines for its use and often tell women to proceed to a health facility to have the abortion completed once they start bleeding. Unless women report misoprostol use, health providers treating these women may have difficulty distinguishing between medication abortions that are in progress and complications due to spontaneous abortions [44]. As a result, our study may have overestimated the number of women treated for abortion complications.

Kinshasa has a relatively high rate of unintended pregnancy, 147 per 1,000 women ages 15–49, compared to other African countries [45]. We estimated that three in five pregnancies are unintended, and about two in five unintended pregnancies are resolved through abortion. These findings demonstrate that many women in Kinshasa are experiencing high levels of unmet need for contraception and are resolving unintended pregnancies through abortion. Kinshasa is a large city; thus, access to contraception, which may be a major problem in rural areas, is likely not an important barrier preventing women who do not want to become pregnant from using modern contraception. Instead, women may have other reasons for not using contraception. Future research should examine reasons for unmet need among women to provide policymakers and program planners evidence based information to design effective policies and programs that address women’s unmet need for contraception. Since unintended pregnancies result from unmet need for contraception and given that unintended pregnancy is the root cause of most abortions, reducing unmet need will not only reduce unintended pregnancy, but will also decrease the number of induced abortions and their related complications, including deaths. Reducing unintended pregnancies and unsafe abortion will also move the DRC closer to achieving Goal 3 (“ensure healthy lives and promote well-being for all at all ages”) of the Sustainable Development Goals (SDGs). By 2030, all countries aim to reduce maternal mortality ratios to less than 70 maternal deaths per 100,000 live births and ensure universal access to sexual and reproductive health services, including family planning [46]. Based on the current situation, the DRC remains far from achieving these targets unless greater effort is made towards reducing maternal mortality and unmet need for contraception. While improving contraceptive uptake can reduce levels of unmet need and unintended pregnancy, increasing access to safe abortion and improving post-abortion care can reduce the negative consequences of unsafe abortion and move the DRC closer to achieving the SDGs [47, 48].

### Limitations

Several limitations of this study are worth mentioning. First, the study team had difficulty compiling a complete and up-to-date list of all health facilities capable of providing PAC in Kinshasa. During the HFS fieldwork, we learned that several facilities were misclassified, either by level of facility (hospital or health center), ownership (public of private/NGO), or both. Given that we had selected 100% of hospitals to be in our sampling frame, it is possible that some hospitals were not captured in our sample. This misclassification could have biased our estimate of the number of induced abortions downwards.

Second, our estimation of the abortion rate is contingent on the number of reproductive-age women living in Kinshasa. The DRC has not conducted a census since 1984; thus, a recent, accurate count of the population does not exist. Instead, we relied on official population estimates produced from population projections of the last census conducted over three decades ago [22].

Third, as is standard practice for the AICM, we assume that women do not seek treatment for first-trimester pregnancy losses and thus only subtract late miscarriages from the total number of treated abortion complications. If a substantial number of women obtain PAC for miscarriages of pregnancies at less than 13 weeks gestation, we may be subtracting too few spontaneous abortion complications from the total number of PAC cases, which would result in overestimates of the number of PAC cases due to induced abortion, as well as the number of abortions.

Fourth, we assumed that all induced abortions were the result of unintended pregnancies. Some of these induced abortions, however, may have been intended at the time of conception. A woman may have later decided to abort the pregnancy because of a change in life circumstances, such as separation from her partner.

Fifth, we did not provide HPS respondents with a formal definition of poor and non-poor women in Kinshasa when we asked respondents to provide responses by poverty status. It is possible that respondents used different definitions of poverty when formulating their responses to our questions.

Finally, our estimate of the annual number of induced abortions in Kinshasa relies heavily on the multiplier that was calculated using data collected from HPS respondents. We selected informants deemed knowledgeable about the practice of abortion and the treatment of abortion complications in Kinshasa. It is important to recognize that respondents’ reports were based on their perceptions of the abortion situation; thus, the accuracy of the multiplier is dependent on the accuracy of their reports.

## Conclusion

Despite the DRC’s restrictive abortion law, thousands of women continue to obtain abortions every year in Kinshasa, of which many are performed unsafely, resulting in severe complications and sometimes death. Our study demonstrates that more effort is needed to reduce the number of unintended pregnancies and unsafe abortions that occur in Kinshasa. Doing so will reduce the number of women who suffer abortion complications and die needlessly from unsafe abortion.

## Supporting information

S1 File Calculation of the multiplier. (DOCX)
