# Supplementary material for: The incidence of induced abortion in Kinshasa, Democratic Republic of Congo, 2016
Source: PLoS One. 2017 Oct 2;12(10):e0184389. doi: 10.1371/journal.pone.0184389 (PMC5624571; doi:10.1371/journal.pone.0184389)
Supplement: S1 File — (DOCX) [file pone.0184389.s001.docx]

**S1 File. Calculation of the multiplier**

Inputs to obtain the multiplier can be expressed as:
D_ijk_ = % of women who receive their abortion from each type of provider, by subgroup and abortion method
S_ijk_ = % of women who experience complications from their abortion, by subgroup, abortion method, and provider
C_i_ = % of women who experience complications from their abortion, among all women in subgroup i
A_ij_ = among all women in subgroup i who have abortions, % who obtain abortion method j
F_ij_ = % of women with complications who receive facility-based treatment, among all women in subgroup i who obtain abortion method j
T_i_ = % of all women having complications who will receive treatment, among all women in subgroup i
P_i_ = % of all women having abortions who will receive treatment, among all women in subgroup i
W_i_ = % distribution of the population of women in Kinshasa, by subgroup
Y = weighted % of women having abortions in Kinshasa who received treatment
M = multiplier

Where:
i=subgroup (poor, non-poor)
j=abortion method (surgical, misoprostol, other)
k=provider (doctor, nurse/midwife, traditional practitioner, pharmacist, self, other untrained person)

To calculate the multiplier:

C_i_ = Σ(D_ijk_ * S_ijk_)
T_i_ = Σ(A_ij_ * F_ij_)
P_i_ = C_i_*T_i_Y = Σ(P_i_*W_i_)
M = 1/Y
